# The stereotypical effect of gendered nicknames on prosocial behavior in online interactions: a chain mediation model

**DOI:** 10.3389/fpsyg.2026.1773532

**Published:** 2026-02-20

**Authors:** Chang You, Hao Shen, Jiaxi Liu, Yun Chen

**Affiliations:** 1 School of Humanities and Management, Hunan University of Chinese Medicine, Changsha, China; 2 School of Psychology, Northwest Normal University, Lanzhou, China

**Keywords:** chain mediation, gendered online nicknames, prosocial behavior, social attractiveness, warmth

## Abstract

**Purpose:**

This study explored how gendered nicknames influence individuals’ prosocial behavior in online interactions and examined the chain mediating roles of warmth and social attractiveness.

**Participants and methods:**

An imaginative context paradigm was used to conduct two experiments in which the participants had to rate their willingness to help users with masculine or feminine nicknames. Experiment 2 further introduced warmth, competence, and interpersonal attraction (social and task) as mediators to test a chain mediation model.

**Results:**

The participants showed a statistically significant greater willingness to engage in prosocial behavior toward users with feminine nicknames. The mediation analysis revealed that warmth and social attractiveness fully mediated the relationship between gendered nicknames and online prosocial behavior, whereas competence and task attractiveness were non-significant.

**Conclusion:**

In the online environment, gendered nicknames shape helping tendencies by influencing cognitive and emotional evaluations. Feminine nicknames evoke warmth and friendliness, enhancing social attractiveness and promoting prosocial intentions. These findings support Eisenberg’s theory of prosocial behavior and highlight how gender stereotypes subtly affect social interactions online.

## 1 Introduction

With the widespread popularity of the Internet, social interaction has gradually transcended the limitations of time and space, expanding from face-to-face communication to text, voice, and video formats. This transformation has greatly enriched people’s social experiences; however, it has also exerted a profound influence on social behavior and patterns of interpersonal communication. A positive change is reflected in the rise of online prosocial behaviors. Against this backdrop, online prosocial behavior demonstrates unique advantages such as rapid dissemination and wide coverage, playing an important role in various contexts and everyday life. As active users of emerging technologies, college students participate widely in online platforms, making their social behaviors particularly susceptible to the shaping influence of the online environment (Jiang and Song, 2023). However, the mechanisms that trigger helping behaviors in online settings remain insufficiently explained, especially with regard to virtual identity cues that may enhance individuals’ willingness to help others.

Online prosocial behavior is formally defined as “voluntary behavior carried out in an electronic context with the intention of benefitting particular others or promoting harmonious relations with others (Pastor et al., 2024, p. 1).” This behavior takes various forms on social networks, such as giving something a “like,” providing instrumental help or information, or offering support in the face of virtual aggression (Pastor et al., 2024). Compared with offline prosocial behavior, it benefits from the Internet’s anonymity and freedom from spatial and temporal constraints, displaying faster dissemination and broader impact (McKenna and Bargh, 2000). For instance, during public events or natural disasters, online fundraising and aid information can quickly attract widespread responses, resulting in the creation of strong social support networks (Sproull, 2011). Such behaviors often accompany self-expression and emotional release, with their social value lying in the promotion of positive interaction and communal mutual assistance (Lin, 2023). Researchers generally agree that online prosocial behavior encompasses both prosocial expression (e.g., posting supportive comments during public incidents) and prosocial action (e.g., calling on organizations to protect consumer rights) (Huang, 2022).

Regarding the measurement of online prosocial behavior, several tools have been developed. For example, some researchers have proposed four dimensions, namely online support, sharing, reminding, and guidance (Zheng et al., 2011), while other researchers have included dimensions such as helping, altruism, cooperation, and reciprocity, and have validated their reliability and validity (Zhang et al., 2022). In the present study, we adopted the College Students’ Online Altruistic Behavior Scale (Zheng et al., 2011) to comprehensively assess participants’ levels of online prosocial behavior.

The theoretical foundation of prosocial behavior is Eisenberg’s integrative model of prosocial behavior (Wang and Pang, 1997). This model addresses the limitations of earlier theories such as group selection and reciprocal altruism by conceptualizing prosocial behavior as a multistage process that includes attending to others’ needs, deciding whether to help, and transforming intention into action. Eisenberg emphasized that cognitive, personality, and emotional factors play critical roles in determining whether an individual engages in prosocial acts. Recent studies have also validated similar emotion–motivation chain mechanisms in adolescents’ prosocial behavior (Hui et al., 2022). Therefore, this study adopted Eisenberg’s analytical framework to examine the cognitive and emotional factors that influence online prosocial behavior. While Eisenberg’s model highlights the internal cognitive and emotional processes, these processes do not work in isolation, they need an external trigger (Eisenberg et al., 2013). In online environments, visual cues are often missing. Instead, users rely on text symbols (Walther, 1992). Therefore, to understand what triggers online prosocial behavior, we must look at the most direct identity cue: the online nickname.

Online nicknames are the most immediate identity markers in virtual environments and often determine other users’ first impressions during the initial stages of interaction. They represent individual identity and convey personality, interests, and emotional attitudes, thereby influencing other users’ initial perceptions and willingness to engage socially. Previous research has shown that although online nicknames differ from real names in naming conventions and usage contexts, both function similarly in cognitive processing: they act as identity cues and play a vital role in social interaction (Stommel, 2007; Yang et al., 2012). Crucially, the definition of gendered nickname needs clarification. It does not rely solely on objective linguistic features. Instead, it refers to subjective social perceptions (Zhou et al., 2021). While nicknames may carry semantic hints, their impact comes from psychological associations. In other words, a gendered nickname is defined by its ability to trigger a masculine or feminine schema in the observer’s mind (Alexander et al., 2021).

Notably, despite the ease with which nicknames can be changed, users tend to retain the same ones. This preference stems from individuals’ tendency to build and sustain social relationships through consistent identity symbols rather than by frequent alterations (Bechar-Israeli, 1995). Nicknames thus play a crucial role in shaping first impressions and promoting social interactions in online environments. Research indicates that self-presentation on social networks often accompanies personal information disclosure, and such identity reinforcement not only enhances self-awareness but may also promote greater prosocial behavior (Valkenburg and Peter, 2011). Specifically, social networking sites enable selective self-presentation, allowing users to present idealized versions of their identities to control or shape how others view them, a process central to impression management (Muyidi, 2025). In this context, the feedback individuals receive through such self-presentation can be regarded as a form of positive social experience, further strengthening their willingness to help others (Nadkarni and Hofmann, 2012). Therefore, as one of the most common and intuitive forms of self-presentation on social networks, nicknames may play a significant role in shaping individuals’ online prosocial behavior.

However, a nickname is not just a simple label, it acts as a social cue (Zhao et al., 2008). When users see a nickname, they quickly form an impression evaluation of the owner. Impression evaluation refers to the process by which individuals form attitudes to and judgments about others based on limited information (Fiske et al., 2002). The stereotype content model (SCM), proposed by Fiske et al. (2002), provides an important theoretical framework for this process, positing that people generally perceive others along two fundamental dimensions: warmth and competence (see also Han, 2021). Of these, warmth is considered the key predictor of prosocial behavior because it directly relates to whether a person is perceived as trustworthy and cooperative. Research has shown that gendered online nicknames can evoke differential evaluative responses. Compared with gender-neutral nicknames, feminine nicknames tend to elicit higher perceptions of warmth and acceptance, although their effect on perceived competence is not statistically significant (Zhang, 2018). This suggests that nicknames, as social identity cues, primarily influence virtual social interactions through perceptions of warmth.

Cuddy and colleagues further proposed the behavior from intergroup affect and stereotypes (BIAS) map, which emphasizes the central role of warmth in predicting helping behavior (Cuddy et al., 2007). Other empirical studies have also demonstrated that positive perceptions of warmth and competence can significantly enhance prosocial intentions (Li, 2022). These findings indicate that in online environments, users’ impressions of nicknames—particularly warmth perception—may serve as key psychological mechanisms that foster prosocial behavior.

According to McCroskey and McCain (1974), interpersonal attraction comprises three dimensions: task attraction, social attraction, and physical attraction. Of these, task and social attraction are most closely associated with cooperation and helping relationships. Warmth and competence impressions play distinct roles in this process: warmth perception tends to enhance social attraction, encouraging individuals to establish friendships and trust, whereas competence perception enhances task attraction, increasing confidence in others’ capabilities (Wojciszke et al., 2009). This relationship can be further elucidated through the lens of social cognitive theory and emotional contagion theory. Social cognitive theory posits that warmth is the primary dimension of social perception, prioritizing the assessment of others’ intentions (Fiske et al., 2007). Perceiving warmth signals safety and benevolence, which fundamentally drives the motivation to approach rather than avoid. Complementarily, emotional contagion theory suggests that the positive affect embodied by warm cues can transfer to the observer (Hatfield et al., 1993). This automatic emotional resonance fosters interpersonal liking and closeness, thereby transforming the perception of warmth into tangible social attraction. Existing evidence indicates that when a target individual is perceived as friendly and likable, observers are more inclined to trust them and experience positive emotions, which in turn lead to greater prosocial behavior (Montoya and Horton, 2012). In online contexts, nicknames, which serve as key cues in forming first impressions, are thus likely to promote prosocial behavior by enhancing social or task attraction if they are perceived as warm or competent, respectively.

More importantly, impression evaluation and interpersonal attraction are not independent processes but are connected through a continuous chain of effects. Warmth and competence perceptions cannot only directly predict prosocial behavior but can also strengthen individuals’ willingness to help through increased interpersonal attraction (Zhang et al., 2021). For instance, the warmth perception evoked by feminine nicknames may enhance social attraction, thereby fostering more mutual assistance and helping behaviors online. Meanwhile, competence perception may operate through task attraction, though its effect on prosocial behavior appears less pronounced than that of warmth. Thus, it can be inferred that the influence of gendered nicknames on online prosocial behavior likely follows a sequential pathway of “impression evaluation → interpersonal attraction.”

In summary, prior research has predominantly focused on offline contexts or single stages of impression evaluation, with little systematic examination of how gendered nicknames affect prosocial behavior in online interactions. Building on the SCM and interpersonal attraction theory, the present study constructed a chain mediation model of “nickname → impression evaluation → interpersonal attraction → online prosocial behavior” to uncover the psychological mechanisms through which virtual identity cues influence online helping behavior. Through this perspective, the study not only fills a gap in existing literature but also provides new theoretical evidence for understanding prosocial behavior in online environments. Accordingly, this study proposes the following hypotheses:

*H1*: The gender of online nicknames significantly affects online prosocial behavior. Compared with users with masculine nicknames (that is, the participants, not the nickname holders), those with feminine nicknames are more likely to exhibit stronger prosocial intentions.

*H2*: Impression evaluation and interpersonal attraction jointly mediate the relationship between gendered nicknames and online prosocial behavior. Specifically, gendered nicknames first influence individuals’ perceptions of warmth and competence, which in turn affect their level of interpersonal attraction toward the nickname holder, ultimately predicting their willingness to engage in online prosocial behavior.

## 2 Experiment 1: the effect of gendered nicknames on prosocial behavior in online environments

### 2.1 Materials and methods

#### 2.1.1 Participants

As calculated by G*Power 3.1, 76 participants (were mostly college students) needed to be recruited for this study (*f* = 0.25, α = 0.05, *p* = 0.95) (Faul et al., 2007). Therefore, a total of 76 participants were initially targeted. However, online questionnaires were circulated to 91 potential participants. From these, 80 valid questionnaires were returned, with an effective response rate of 88%. The sample comprised 30 males and 50 females, with an average age of 22.41 (*SD* = 1.78) years and the average number of years of using the Internet was 10.65 (*SD* = 3.03). The participants were assumed to be experienced Internet users familiar with the concept of online nicknames. This study was approved by the Ethics Committee of Northwest Normal University (Approval No. 2025329). Before filling out the questionnaire, participants were fully informed of the survey’s objectives, relevant precautions, privacy protection measures, as well as the management and storage of the collected data. Voluntary completion and submission of the online questionnaire by participants was regarded as their provision of informed consent, and they were entitled to withdraw from the study at any time.

#### 2.1.2 Design

A one-way within-subjects experimental design was used, with the gendered online nickname (masculine or feminine) as the within-subjects variable and the participants’ willingness to implement web support, mentoring, sharing, and reminders for users as the dependent variables.

#### 2.1.3 Materials

The method for selecting online nicknames was adapted from a previous study (Li and Wu, 2013). Two sources of online nicknames were used. The first involved selecting 20–50 pure Chinese character-based nicknames from each participant’s friend list and various group chats without any discrimination. The second source was major online platforms. For 1 week, at 12:30 p.m. and 7:00 p.m. each day, pure Chinese character-based nicknames appearing in hot searches were collected, ultimately forming a nickname library of 1,000 nicknames. After excluding those that did not meet the study criteria, 80 nicknames were randomly selected for inclusion in the study.

The 80 online nicknames were then evaluated by 30 participants on a scale of 1–7 for gendering and familiarity, with 1 being very non-conforming and 7 being very conforming. Genderedness was evaluated using a single-score ranking and two-dimensional evaluation method, followed by a paired-samples *t*-test. The selection involved two steps. First, the scores for masculinity and femininity were sorted separately, and the top 15 nicknames were selected for each category by the gender rating score. Second, we calculated the difference score for each nickname. The masculinity score was subtracted from the femininity score. The range for masculinized nicknames was [0, 6), and the range for feminized nicknames was (–6, 0]. The nicknames were then ranked by the difference score, and the mean of the two rankings was calculated to identify the top five masculinized and feminized nicknames, totaling 10 nicknames. Gender-neutral or non-binary nicknames were excluded from the current design to minimize perceptual ambiguity (Alexander et al., 2021).

A paired-samples *t*-test was conducted, yielding significant results for the 10 nicknames. Feminine nicknames received statistically significantly higher femininity scores (*M* = 6.27, *SD* = 0.47) than masculinity scores (*M* = 2.26, *SD* = 0.81), *t*(29) = 19.98, *p* < 0.001. Similarly, masculine nicknames received statistically significantly higher masculinity scores (*M* = 6.50, *SD* = 0.44) than femininity scores (*M* = 1.91, *SD* = 0.68), *t*(29) = –24.84, *p* < 0.001. There was no statistically significant difference in familiarity scores between feminine (*M* = 4.13, SD = 1.25) and masculine (*M* = 4.30, *SD* = 1.25) nicknames, *t*(29) = –1.15, *p* = 0.258, which ruled out the effect of familiarity in the subsequent research.

The online prosocial behavior measure was adopted from the Online Altruistic Behavior Scale for College Students (Zheng et al., 2011). The scale divides online prosocial behavior into four dimensions: online support, sharing, reminders, and guidance. These dimensions align with the purpose of this study. An example question was: “How willing are you to guide ‘Lin Shuya’ on how to use the Internet better?” (1 = very reluctant, 7 = very willing). The alpha coefficient of the scale in the original study was 0.94, and the alpha coefficients of the dimensions ranged from 0.80 to 0.88. In this study, the alpha coefficient of the scale was 0.99, and the alpha coefficients of the dimensions ranged from 0.83 to 0.86.

#### 2.1.4 Procedures

This study used an imaginative context paradigm with experimental materials adapted from the contextual guides of Study 2 in Bao et al. (2016). An experimental questionnaire was developed using SoJump (a survey research company in China).^1^ Before beginning the experiment, the researcher explained the purpose and process of the study to the participants in detail. The participants were informed that the study aimed to explore the impact of online nicknames on their willingness to engage in online prosocial behavior. Special attention was paid to anonymity and confidentiality to ensure that the participants could freely share their true thoughts and feelings.

Subsequently, the participants were introduced to the specific context of the experiment in the questionnaire. They were told that 10 Internet users would be randomly matched to them for an online conversation. Owing to the limited information, the participants could only see the online nicknames of these users and had no access to other personal details. This setup was designed to simulate a real online social environment, allowing participants to evaluate and judge their online peers based solely on their nicknames.

After the experimental context was explained, the participants were asked to rate the willingness of the 10 internet users to engage in online prosocial behavior. Ratings were made on a scale of 1–7 (1 = very reluctant, 7 = very willing). In this study, online prosocial behavior comprised four dimensions—online support, reminders, guidance, and sharing—with three questions for each dimension to comprehensively assess participants’ willingness to engage in prosocial behavior. The researchers asked the participants to evaluate each of the fictitious Internet user’s willingness to engage in prosocial behavior in each of the four dimensions. Finally, the overall mean was calculated as an evaluation index of the participants’ willingness to implement prosocial behavior online.

### 2.2 Results

#### 2.2.1 Manipulation check

The results of the paired-samples *t*-test for all 10 nicknames in terms of gendering were statistically significant. The feminization evaluation scores (*M* = 5.97, *SD* = 0.63) for the five feminine nicknames were statistically significantly higher than the masculinization scores (*M* = 2.53, *SD* = 0.91), *t*(79) = 21.47, *p* < 0.001. Similarly, the masculinization evaluation scores (*M* = 6.23, *SD* = 0.55) for the five masculine nicknames were statistically significantly higher than the feminization scores (*M* = 2.23, *SD* = 0.86), *t*(79) = –27.67, *p* < 0.001. There was no statistically significant difference in the familiarity scores between feminine (*M* = 3.81, *SD* = 1.17) and masculine (*M* = 3.91, *SD* = 1.21) nicknames, *t*(79) = –0.23, *p* = 0.305.

#### 2.2.2 Descriptive statistics and ANOVA results

The results of the descriptive statistics of the masculine and feminine nicknames and the two dimensions of online prosocial behavior revealed that the scores and total scores of the dimensions of feminine nicknames were higher than those of the dimensions of masculine nicknames. The highest preference scores were obtained for implementing guidance for masculine nicknames (*M* = 5.05, *SD* = 1.50) and reminders for feminine online nicknames (*M* = 5.50, *SD* = 1.20). Table 1 presents these results.

**TABLE 1:** Descriptive statistical results of the gendered online nickname implementation of the online prosocial behavior Questionnaire [M, (SD)].

Variables	Masculine online nicknames (*n* = 80)	Feminine online nicknames (*n* = 80)
Online support	4.63 (1.71)	5.38 (1.22)
Online guidance	5.05 (1.50)	5.54 (1.16)
Online sharing	4.93 (1.59)	5.44 (1.19)
Online reminder	5.00 (1.55)	5.50 (1.20)
Online prosocial behavior total score	19.61 (4.68)	21.77 (3.02)

**FIGURE 1:**
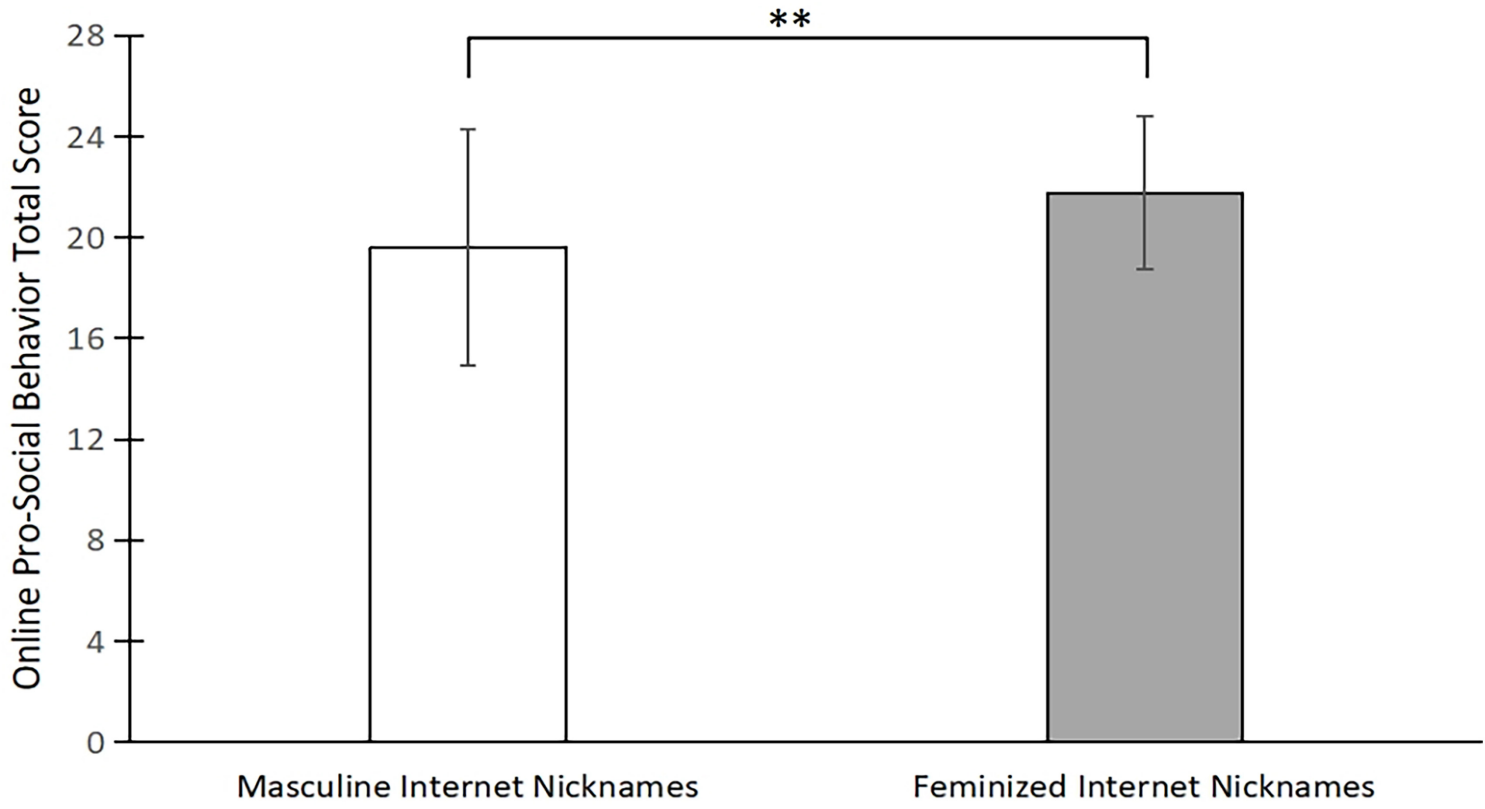
Inferential statistical results of online prosocial behavior. **p < 0.01.

A one-way repeated measures analysis of variance (ANOVA) was conducted on online prosocial behaviors and revealed a statistically significant difference in participants’ willingness to implement such behaviors for different gendered nicknames, *F*(1,79) = 95.65, *p* < 0.001, η^2^ = 0.55. Furthermore, the participants were more willing to help individuals who adopted feminine nicknames (*M* = 21.77, *SD* = 3.02) than those who adopted masculine nicknames (*M* = 19.61, *SD* = 4.68). These results are shown in Figure 1.

## 3 Experiment 2: the Chain mediating roles of impression evaluation and interpersonal attraction in the effect of gendered nicknames on prosocial behavior online

### 3.1 Materials and methods

#### 3.1.1 Participants

As calculated by G*Power 3.1, 76 participants needed to be recruited for this study (*f* = 0.25, α = 0.05, *p* = 0.95) (Faul et al., 2007). Therefore, a total of 76 participants were initially targeted. However, a total of 82 valid participants were recruited, comprising 33 males and 49 females, with a mean age of 21.33 (*SD* = 2.86) years and the average number of years of using the Internet was 8.26 (*SD* = 3.14). The participants were assumed to be experienced Internet users familiar with the concept of online nicknames. This study was approved by the Ethics Committee of Northwest Normal University (Approval No. 2025329). And the participants were compensated after completing the study.

#### 3.1.2 Design

A one-way within-subjects experimental design was used. Online nicknames (gendered: masculine and feminine) were the within-subject variable, and the dependent variable was the participants’ willingness to engage in prosocial behavior toward the user. The mediating variable was the participants’ impression evaluation score of the user, including the dimensions of warmth and competence, and the interpersonal attraction score, including the dimensions of social and task attraction.

#### 3.1.3 Materials

Online nicknames and prosocial behavior materials were the same as those used in Experiment 1. Trait words for the warmth and competence dimensions in the impression evaluation measure were adapted from Fiske et al. (2002). In this study, non-negative nicknames were selected; thus, positive trait words were used. Three positive traits were used to measure participants’ warmth evaluation of the nickname “MeiGuiTa,” examining the extent to which they believed that “MeiGuiTa” was “helpful,” “sincere and trustworthy,” and “friendly and tolerant” (1 = strongly disagree, 7 = strongly agree). Three positive traits were used to measure participants’ competence evaluation of the online nickname, asking to what extent they believed that “MeiGuiTa” was “intelligent and knowledgeable,” “efficient in completing tasks,” and “creative” (1 = strongly disagree, 7 = strongly agree). In this study, the internal consistency reliability for the warmth dimension trait words was 0.84, and that for the competence dimension trait words was 0.81.

The interpersonal attraction measure was adapted from McCroskey’s Interpersonal Attraction Scale (McCroskey et al., 2006). The Chinese version of this scale has been tested for reliability and validity with good results (Bao et al., 2016). The task attraction dimension consisted of four items, one of which was reverse scored. An example item was: “Do you feel confident that ‘Lin Shuya’ can complete the work?” (1 = strongly disagree, 7 = strongly agree). The social attraction dimension also comprised four items, one of which was reverse scored. An example item was: “Do you find ‘Lin Shuya’ easy to get along with?” (1 = strongly disagree, 7 = strongly agree). In this study, the internal consistency reliability for the task and social attraction dimensions were 0.80 and 0.86, respectively.

#### 3.1.4 Procedure

This study used an imaginative context paradigm with experimental materials adapted from the contextual guides of Study 2 in Bao et al. (2016). As with Experiment 1, the experimental procedure was developed using SoJump. Before the experiment began, the participants were informed that they would be involved in a study on online nicknames. They were then introduced to 10 Internet users who were randomly matched to them for an online conversation. Owing to the limited information, only the nicknames of these users were shown to the participants. The participants rated the 10 Internet users on a scale of 1–7 based on two dimensions: impression evaluation and interpersonal attraction. The mean rating for each dimension was then calculated and used as an evaluation index.

### 3.2 Results

#### 3.2.1 Descriptive statistics and correlation analysis

The results of the descriptive statistics and correlation analyses of the variables are shown in Table 2. Gendered nicknames were the categorical variable, where 1 represented feminine nicknames and 2 represented masculine nicknames. Table 2 also presents the results of the correlation analyses. The findings indicated that the data from this study were suitable for the next step in the data analysis.

**TABLE 2:** Correlation coefficients of the variables (*n* = 82).

Variables	M	SD	1	2	3	4	5
1. Gendered online nicknames				0.75**	0.75**	0.82**	0.77**
2. Warmth dimension	5.19	0.86	–0.30**				
3. Competence dimension	4.9	1.05	–0.11				
4. Social attractiveness dimension	5.04	1.12	–0.36**	0.83**			
5. Task attractiveness dimension	5.06	0.90	–0.15	0.77**	0.84**		
6. Online prosocial behavior	10.69	2.07	–0.27**	0.77**	0.73**	0.86**	

**p < 0.01.

#### 3.2.2 Inferential statistical results of impression evaluation

A one-way repeated measures ANOVA was conducted on the means of the impression evaluation dimensions for different gendered nicknames. There was a statistically significant difference in the scores of different gendered nicknames for the warmth dimension, *F*(1, 81) = 31.80, *p* < 0.001, η^2^ = 0.28. The warmth scores of feminine nicknames (*M* = 5.45, *SD* = 1.04) were statistically significantly higher than those of masculine nicknames (*M* = 4.93, *SD* = 1.49). Furthermore, there was no statistically significant difference in the scores of different gendered nicknames for the competence dimension. However, the competence scores of feminine nicknames (*M* = 5.02, *SD* = 1.44) were higher than those of masculine nicknames (*M* = 4.79, *SD* = 1.62).

#### 3.2.3 Inferential statistical results of interpersonal attraction

**FIGURE 2:**
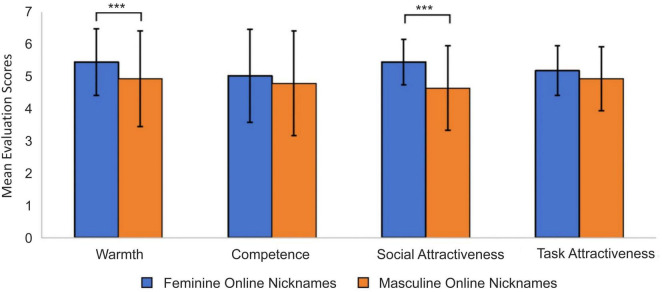
Inferential statistical results of impression evaluation and interpersonal attraction. ^***^p < 0.001.

A one-way repeated measures ANOVA on the means of the interpersonal attraction dimensions for the gendered nicknames revealed a statistically significant difference in the scores of the social attraction dimension, *F*(1, 81) = 53.08, *p* < 0.001, η^2^ = 0.40. The social attraction scores for feminine nicknames (*M* = 5.45, *SD* = 0.70) were statistically significantly higher than those for masculine nicknames (*M* = 4.64, *SD* = 1.31). There was no statistically significant difference in the scores of different gendered nicknames for the task attractiveness dimension. But the task attractiveness scores for feminine nicknames (*M* = 5.18, *SD* = 0.77) were higher than those for masculine nicknames (*M* = 4.93, *SD* = 0.99). The results are shown in Figure 2.

#### 3.2.4 Mediation effect analysis

**FIGURE 3:**
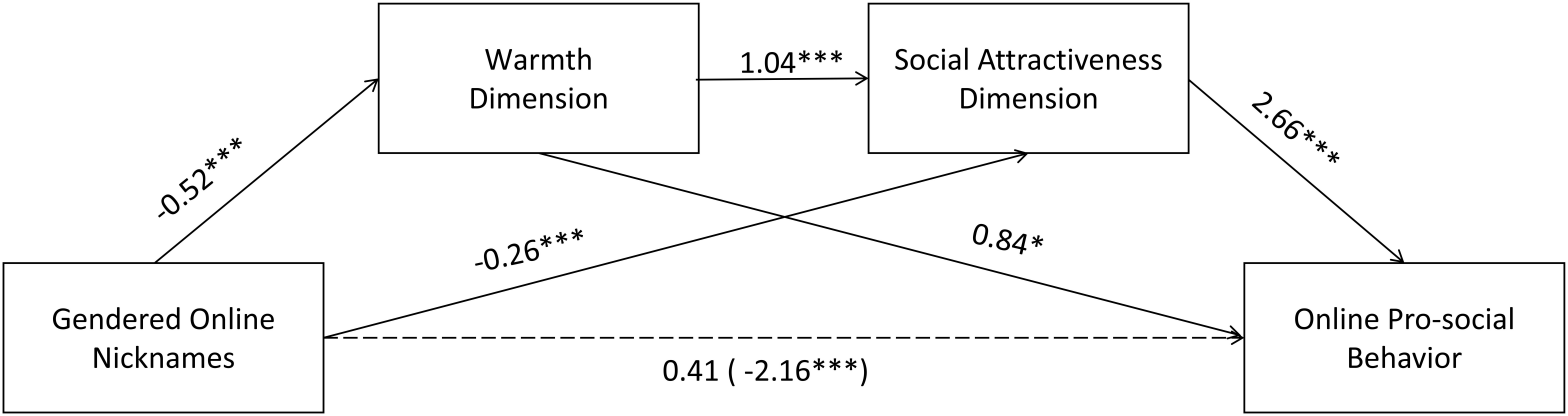
Chain mediating role of impression evaluation and interpersonal attraction in the effect of gendered online nicknames on online prosocial behavior. *p < 0.05, ^***^p < 0.001.

To explore the mediating role of impression evaluation in the process of gendered nicknames influencing online prosocial behavior, the study used a mediation model with gendered nicknames as the independent variable (gendered nicknames as the grouping variable, in which 1 is feminine nicknames and 2 is masculine nicknames), impression evaluation as the mediator, and online prosocial behavior as the dependent variable. The mediator model was tested using the Hayes Process model 6 (v3.3), and the sampling was repeated 5,000 times to calculate the bias-corrected bootstrap 95% confidence intervals (see Figure 3 and Tables 3, 4).

**TABLE 3:** Chain mediating role of impression evaluation and interpersonal attraction.

Variables	Equation 1 (dependent variable: warmth dimension)			Equation 2 (dependent variable: social attractiveness dimension)			Equation 3 (dependent variable: online prosocial behavior)		
	β	SE	*t*	B	SE	*t*	β	SE	*T*
Gendered Online nicknames	–0.52	0.13	–4.05***	–0.26	0.10	–2.63**	0.41	0.34	1.2
Warmth dimension				1.04	0.06	17.89***	0.84	0.34	2.49*
Social attractiveness dimension							2.66	0.27	10.04***
*R* ^2^	0.09			0.71			0.75		
*F*	16.42***			195.59***			162.39***		

*p < 0.05, **p < 0.01, and ***p < 0.001.

**TABLE 4:** Chain mediation effect analysis of impression evaluation and interpersonal attraction.

Effect	Trails	Efficiency value	SE	95% CI		Relative mediation effect (%)
				Low	High	
Total effect		–2.16	0.62	–3.38	–0.95	1.00
Direct effect		0.41	0.34	–0.26	1.09	
Intermediary effect	Gendered online nicknames → warmth dimension → online prosocial behavior	–0.44	0.34	–1.13	0.24	0.17
	Gendered online nicknames → social attractiveness dimension → online prosocial behavior	–0.70	0.26	–1.22	–0.20	0.27
	Gendered online nicknames → warmth dimension → social attractiveness dimension → online prosocial behavior	–1.44	0.45	–2.47	–0.66	0.56

## 4 Discussion

This study extends previous research on prosocial behavior by examining whether gendered nicknames affect the implementation of online prosocial behavior from an evaluator’s perspective.

### 4.1 Gendered nicknames as triggers for online helping behavior

The core finding of this study is that in computer-mediated communication (CMC) environments with relatively scarce information, gendered nicknames serve as powerful initial cues that significantly influence others’ willingness to engage in online prosocial behavior. Specifically, users with feminine nicknames were more likely to receive assistance than those with masculine nicknames. This finding confirms that gender, as a key variable influencing prosocial behavior, extends its effect from offline to online contexts. This aligns with the classic findings of Eagly and Crowley (1986), who observed that women receive more help than men in offline settings. It also resonates with Hirani’s perspective that gender serves as an internal factor influencing prosocial behavior (Hirani et al., 2022). Furthermore, recent research highlights that while the Internet’s anonymity provides freedom, the disclosure of identity cues is closely linked to strategies aimed at maintaining reputation and social perception, suggesting that specific identity markers significantly shape online behavioral norms (Muyidi, 2025). Building on this theoretical foundation, this study goes beyond observing direct effects to reveal the underlying multi-step mediating mechanism: gendered nicknames influence others’ evaluative impressions of the nickname holder (particularly on the warmth dimension), thereby enhancing interpersonal attraction (especially social attractiveness), which ultimately translates into differences in willingness to engage in prosocial behavior.

### 4.2 Stereotype activation and the “warmth priming” effect

During the initial stages of online interaction, users rely heavily on limited cues to quickly form an impression of strangers. Gendered nicknames are not neutral labels but serve as highly efficient cognitive heuristics that promptly activate gender-related stereotypical associations. According to the SCM, feminine names are typically associated with communal traits such as warmth and friendliness, while masculine names are linked to agentic traits such as competence and decisiveness (Zuo et al., 2021).

The findings of this study are highly consistent with the SCM’s primacy of warmth principle, which posits that when forming social judgments and behavioral tendencies, evaluations of others’ intentions (i.e., warmth) take precedence over assessments of their abilities (Zuo et al., 2020). Recent research provides robust cross-domain evidence supporting this principle. For example, a study on destination brand perception found that warmth—rather than competence—served as the key mediating variable between gendered brand imagery and tourists’ identification with the destination (Hamdy et al., 2024). Similarly, in advertising, “soft-sell” voice styles were perceived as warmer than “hard-sell” ones, and this warmth perception directly drove consumer preference (Desmarais et al., 2025).

In this study, participants rated feminine nicknames significantly higher on the warmth dimension than masculine ones, while no significant difference was found regarding the competence dimension. This pattern precisely illustrates that in social contexts, perceived warmth carries far greater weight than competence as the primary criterion for evaluating others’ intentions.

### 4.3 From impression to attraction: warmth as a catalyst for interpersonal attraction

When the mediating chain shifts from cognitive evaluation (impression) to emotional response (attraction), the findings demonstrate that the “high warmth” perception triggered by feminine nicknames directly fosters social attraction. According to interpersonal attraction theory, traits such as friendliness and sincerity—specific manifestations of warmth—are central to motivating individuals to initiate interaction and establish interpersonal relationships (McCroskey and McCain, 1974).

This core mechanism receives strong support from a parallel model proposed in recent human–computer interaction research. A 2025 study systematically examined how the characteristics of chatbots—such as ChatGPT—shape user engagement, and identified social attractiveness and perceived affinity as two independent exogenous social factors influencing human–chatbot relationships. Structural equation modeling revealed that both forms of social attributes significantly enhanced users’ parasocial interaction and their perceived emotional support during interactions with the chatbot. Further analyses showed that these relational responses served as key mediators linking chatbot attributes to critical user behaviors, including usage intention and media dependence. Taken together, the findings suggest that social attributes promote emotional bonding with the agent, which subsequently drives sustained use and psychological reliance on the medium (Zhang et al., 2025).

Furthermore, this study further identified that feminine nicknames significantly increased perceived warmth. And the heightened warmth then fostered stronger social attraction (Zhang et al., 2021). Finally, this social attraction directly drove the willingness to help. Notably, the direct path from warmth perception to prosocial behavior was not significant. This finding is crucial. It indicates that warmth alone does not automatically trigger helping. Instead, its influence is fully transmitted through social attraction. In other words, users are helped not just because they seem warm, but because that warmth makes them attractive as social partners.

This provides a parallel model for the current findings: when a user has a feminine nickname and is perceived as “warm,” this cognitive evaluation enhances observers’ social attraction toward the user, and such positive emotional connection ultimately drives their willingness to engage in helping behaviors.

### 4.4 From attraction to action: the transformation of emotion, motivation, and behavior

The final stage of this study confirms that social attraction fully mediates the effect of gendered nicknames on online prosocial behavior. The underlying logic suggests that the positive emotional states generated through interpersonal attraction serve as powerful motivators for helping behavior. Eisenberg’s theory of prosocial reasoning proposes that empathy and perspective-taking are key catalysts for prosocial behavior (Wang and Pang, 1997). When individuals experience social attraction toward someone, they are more likely to feel empathy, thereby lowering the psychological threshold for offering help.

Recent neuroscience findings provide biological evidence for this emotion-to-action transformation. A functional near-infrared spectroscopy (fNIRS) study revealed that positive emotions directly promote prosocial behavior by activating brain regions associated with executive control—the right dorsolateral prefrontal cortex and the medial prefrontal cortex (Nie et al., 2025). These findings suggest that positive emotions elicited by high warmth and strong social attraction can physiologically facilitate altruistic decision-making.

However, the phenomenon of “greater willingness to help users with feminine nicknames” warrants careful interpretation. This behavioral pattern may partly result from the women-are-wonderful effect, which posits that women are generally evaluated more positively than men on communal traits (Eagly and Mladinic, 1994). A deeper explanation, however, may involve benevolent sexism (BS)—an attitude that portrays women as needing protection, offering seemingly positive regard while ultimately constraining their autonomy (Kasof, 1993). In our study, feminine nicknames evoked high warmth perceptions. This aligns with the benevolent sexism framework, where women are stereotyped as warm and friendly. Crucially, this high warmth predicted stronger helping intentions (Ramos et al., 2018). Recent research has highlighted that BS tendencies are maintained through social norms and are closely associated with dependency-oriented helping—a form of assistance that reinforces traditional gender roles (Cao and Cao, 2018; Feng, 2023). Notably, our results showed that participants were most willing to provide online guidance to users with feminine nicknames. This dimension, which involves instructing others on how to act, typically implies a competence gap and reinforces a hierarchy where the helper assumes the role of an expert (Nadler, 2002; Zheng et al., 2011). Thus, the increase in prosocial behavior observed in this study may represent a complex combination of genuine empathy and the unconscious reinforcement of restrictive gender stereotypes. Future research should examine whether feminine nicknames specifically elicit dependency-oriented help, thus clarifying if such assistance restricts their autonomy.

However, we must also acknowledge an alternative explanation that warmth perceptions may be the primary driver. According to the SCM, warmth signals cooperative intent and safety. This signal naturally elicits social approach behaviors such as helping (Fiske et al., 2002). In this view, participants may have helped users with feminine nicknames simply because they perceived them as more likable and trustworthy (Cuddy et al., 2007). They did not necessarily view them as weak or needing protection. Distinct from the restrictive nature of benevolent sexism, this mechanism reflects a general social preference for friendly partners. Therefore, the observed prosocial behavior likely stems from a dual process. The general appeal of warmth motivates the willingness to engage. Meanwhile, gender stereotypes shape the specific form of assistance such as dependency-oriented guidance. Future research should further disentangle these two mechanisms.

### 4.5 Theoretical contributions, limitations, and future directions

Theoretically, this study provides new empirical evidence supporting the applicability of Eisenberg’s theory of prosocial behavior in the digital age. It also demonstrates how the SCM framework can explain the full process linking micro-level online cues to macro-level social behavior, addressing a critical gap in existing literature regarding how social media-specific factors shape prosocial behavior.

Several limitations should be acknowledged. First, college students were selected as the primary sample in this study due to their high level of online activity. However, relying solely on this demographic group as research participants will inevitably limit the generalizability of the findings. Future research should recruit more diverse populations (e.g., adolescents and working professionals) to improve the ecological validity of the study. Second, this research examined only specific types of nicknames, excluding gender-neutral and non-binary forms. Earlier studies suggested that gender-ambiguous names were often perceived as male by default, leading to lower evaluations (Kasof, 1993). However, as societal gender norms evolve and non-binary identities become increasingly recognized, the social meanings of traditional gender cues such as names are being reinterpreted. Research has shown that contemporary youth emphasize fluidity and self-definition in gender identity, while gender expression in online interaction exhibits unprecedented diversity (Allen et al., 2022; Dorn et al., 2025). These trends suggest that the long-held assumption that “gender-ambiguous names are defaulted as male” requires critical reexamination. Third, this study was conducted in a collectivist culture where communal traits and interpersonal harmony are highly prioritized (Cuddy et al., 2015), a context that may amplify the positive effects of feminine nicknames on warmth perceptions. Consequently, future cross-cultural research is necessary to determine if these gendered mechanisms operate similarly in individualistic societies where agentic traits are often more valued.

Additionally, several potential confounding variables related to participant characteristics were not fully controlled. On the one hand, participants’ personal gender role attitudes were not measured, yet these individual differences could moderate how users perceive gendered nicknames. On the other hand, this study did not consider the gender of the helpers themselves, even though research indicates potential preferences for same-sex or opposite-sex helping (Fiala et al., 1999).

Finally, future research should include a key moderating variable—social value orientation (SVO). SVO assesses the extent to which individuals care about others’ welfare and is typically categorized as prosocial or proself (Murphy et al., 2011). A 2025 study confirmed that SVO significantly moderates the relationship between pro-environmental behavior (a type of prosocial behavior) and its outcomes: individuals with prosocial orientations report higher quality of life when engaging in pro-environmental actions (Purnama et al., 2025). Therefore, future research should explore whether the effect of gendered nicknames on helping behavior is moderated by SVO—that is, whether the effect is stronger among individuals with prosocial orientations.

## 5 Conclusion

This study clarifies an important social–cognitive pathway, showing how subtle gender cues such as nicknames can influence prosocial behavior—a broader social outcome—in computer-mediated interactions. The findings indicate that feminine nicknames are more effective in eliciting helping intentions than masculine ones. Importantly, the chain-mediated model shows that gendered nicknames act as initial cues that trigger distinct impression evaluations focused on the “warmth” dimension. This warmth-based cognitive judgment subsequently leads to emotional interpersonal attraction. Ultimately, it is this emotional attraction—rather than the nickname itself—that accounts for the observed differences in subsequent prosocial behavior. These findings offer practical implications for online environments. First, social media platforms should optimize interface designs. Features that allow flexible self-presentation can help users manage social impressions beyond traditional gender stereotypes. Second, Internet literacy education is essential. Curricula should include awareness training regarding unconscious gender bias in online interactions. This helps individuals recognize how subtle cues like nicknames influence their behavior. Finally, interventions aimed at promoting equality are needed. Encouraging users to provide help based on actual needs rather than social attractiveness can foster a more inclusive online community.

## Data Availability

The original contributions presented in this study are included in this article/Supplementary material, further inquiries can be directed to the corresponding author.

## Footnotes

1
https://www.wjx.cn/
