# Supplementary material for: The stereotypical effect of gendered nicknames on prosocial behavior in online interactions: a chain mediation model
Source: Front Psychol. 2026 Feb 20;17:1773532. doi: 10.3389/fpsyg.2026.1773532 (PMC12964138; doi:10.3389/fpsyg.2026.1773532)
Supplement: Supplementary file 1 [file Table_1.docx]

**Table S1**

The Final 10 Gendered Nicknames Used in the Experiments

| Category | Chinese Nickname | Romanization | English Meaning / Connotation |
| --- | --- | --- | --- |
| Feminine | 暖暖兮 | Nuan Nuan Xi | "Nuan" means warm; "Xi" is a soft particle. Implies cuteness and warmth. |
|  | 林树娅 | Lin Shu Ya | "Ya" is a typical character used in female names. Sounds gentle. |
|  | 艾露莎 | Ai Lu Sha | Transliteration similar to "Elsa"; distinctly Western feminine style. |
|  | 月慕曦 | Yue Mu Xi | "Yue" (Moon), "Mu" (Admire), "Xi" (Dawn light). Poetic and graceful. |
|  | 简曦童 | Jian Xi Tong | "Xi" (Dawn), "Tong" (Child). Implies innocence and youthfulness. |
| Masculine | 战神琛 | Zhan Shen Chen | "Zhan Shen" means God of War. Implies aggression and dominance. |
|  | 黄振雄 | Huang Zhen Xiong | "Zhen" (Revitalize), "Xiong" (Hero/Male). Traditional masculine name. |
|  | 古惑仔 | Gu Huo Zi | "Young and Dangerous" (referring to tough guys/gangsters). Implies roughness. |
|  | 唐建辉 | Tang Jian Hui | "Jian" (Build), "Hui" (Splendor). Traditional masculine name implying success. |
|  | 赵汉杰 | Zhao Han Jie | "Han" (Man/Han Dynasty), "Jie" (Hero/Outstanding). Implies strength. |
